# Cancer-specific binary expression system activated in mice by bacteriophage HK022 Integrase

**DOI:** 10.1038/srep24971

**Published:** 2016-04-27

**Authors:** Amer Elias, Itay Spector, Ilana Sogolovsky-Bard, Natalia Gritsenko, Lene Rask, Yuli Mainbakh, Yael Zilberstein, Ezra Yagil, Mikhail Kolot

**Affiliations:** 1 Department of Biochemistry and Molecular Biology, Tel-Aviv University, Tel-Aviv 69978, Israel; 2 Department of Oncology 54O5, Herlev Hospital, University of Copenhagen, Herlev Ringvej 75, 2730 Herlev, Denmark; 3 Department of Neuroscience and Pharmacology, University of Copenhagen and Department of Clinical Neurophysiology, Rigshospitalet - Glostrup, Entrance 3, 9th floor, Nordre Ringvej 57, 2600 Glostrup, Denmark

## Abstract

Binary systems based on site-specific recombination have been used for tumor specific transcription targeting of suicide genes in animal models. In these binary systems a site specific recombinase or integrase that is expressed from a tumor specific promoter drives tumor specific expression of a cytotoxic gene. In the present study we developed a new cancer specific binary expression system activated by the Integrase (Int) of the lambdoid phage HK022. We demonstrate the validity of this system by the specific expression of a luciferase (*luc)* reporter in human embryonic kidney 293T (HEK293T) cells and in a lung cancer mouse model. Due to the absence viral vectors and of cytotoxicity the Int based binary system offers advantages over previously described counterparts and may therefore be developed into a safer cancer cell killing system.

Site-specific recombinases are widely used for genome manipulations by catalysis of site-specific recombination reactions (SSR) between two defined DNA sequences of ~20–200 base pairs (bp) functioning as recombination sites (RSs)^1^,^2^,^3^,^4^,^5^,^6^. SSR reactions result in integration, excision or inversion, depending on the location and relative orientation of the RSs. A widely used SSR-based technique is the recombinase-mediated-cassette-exchange (RMCE) reaction allowing clean exchange of an existing gene by a gene of interest^7^. SSR systems that are currently used to manipulate genomes of higher organisms include those of the bacteriophage P1 Cre/*lox*, the baker yeast Flp/*frt* and the bacteriophage φC31 Int/*att*. The recombinases of the former two systems belong to the tyrosine recombinase family and of the latter system to the serine recombinase family^8^.

SSR systems are also used in binary systems for cell and gene specific expression that are based on two DNA modules. One module carries a recombinase substrate comprising a target gene silenced by a transcription terminator placed between two RSs. The second module carries the recombinase gene controlled by a cell type specific promoter. This promoter targets the expression of the SSR to the desired cells where it excises the terminator and thus activates the expression of the gene of interest^9^,^10^,^11^.

Coliphage HK022 encodes an Integrase recombinase (Int) belonging to the tyrosine integrase family that catalyzes the integration of the phage genome into its RS on the *Escherichia coli* chromosome as well as its excision from the *E. coli* chromosome. In these regards it acts very much like the well-documented coliphage λ Integrase^12^. Int targets a 21 bp long bacterial RS termed *attB* or *BOB*′ to indicate its composition of a central 7 bp overlap region (*O*) and the two flanking incomplete inverted repeats of 7 bp (*B* and *B*′). *O* is the site of DNA exchange while *B* and *B*′ are Int binding sites. The phage recombination site termed *attP* is ~200 bp long. It contains a 21 bp core (*COC*′) that is similar to *attB* and is flanked by two long arms (*P* and *P*′) that carry additional Int binding sites and others that bind accessory proteins required for the SSR reactions. The phage integration reaction occurs between *attP* and *attB.* It yields two hybrid recombination sites flanking the integrated prophage termed *attL* (*BOP*′) and *attR* (*POB*′). The reverse prophage excision reaction occurs between the *attL* and *attR* sites and restores the original *attP* and *attB*^13^. We have previously applied Int for genome manipulation in plants, cyanobacteria and human cells and have shown that the accessory proteins that are required in the *E. coli* host are dispensable in the eukaryotic milieu^14^,^15^,^16^,^17^,^18^. In the present study we developed a cancer specific binary cell expression system that is based on the *hTERT* promoter. *hTERT* serves as the catalytic reverse transcriptase subunit of telomerase and is specifically expressed in immortal and cancer cells^19^,^20^. In this binary cell expression system *hTERT*-promoted Int specifically activates a *luc* reporter in immortalized human embryonic kidney 293 cells (HEK293T) and in mouse lung tumors originating from injected Lewis lung carcinoma line 1 (LLC1). This cancer specific expression system offers certain advantages over those previously described^9^,^10^,^11^ and may be applied to express toxins specifically in cancerous tumors.

## Results

### Cancer specific *luc* reporter expression mediated by an Int-based binary system

**Figure 1:**
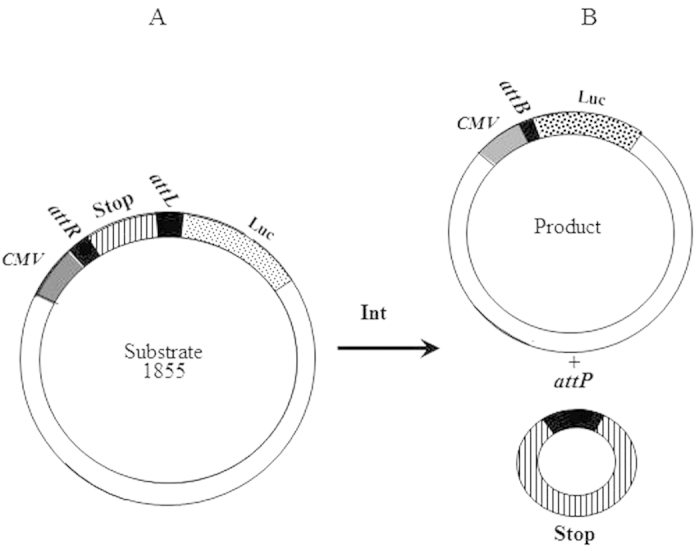
Scheme of the Int-catalyzed binary system to express a *luc*-reporter in cancer cells. (**A**) The recombination substrate carrying a silenced *luc* reporter separated from its *CMV* promoter by a transcription terminator (Stop) flanked by *attR* and *attL* recombination substrates. (**B**) The recombination products generated by Int: The activated *luc*-reporter separated from its *CMV* promoter by the small bacterial recombination site *attB* (top), and the excised non-replicative Stop (bottom).

Two plasmids were constructed to generate a cancer specific binary cell expression system activated by Int. One plasmid expresses the *int* gene under the control of the cancer specific promoter *hTERT*^19^,^20^ or by the constitutive cytomegalovirus (*CMV*) promoter as control. The other carries a *luc* gene separated from a *CMV* promoter by a transcription terminator (Stop) flanked by tandem *attR* and *attL* recombination sites (Fig. 1). Int-catalyzed *attR* x *attL* recombination excises Stop allowing expression of the *luc* reporter.

### Validation that the *luc* reporter cancer specific binary cell expression system is activated by Int in HEK293T cells

**Figure 2:**
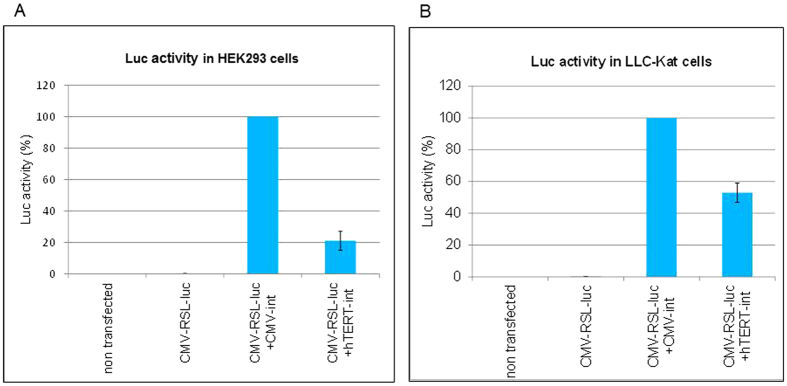
Luc activity assays of the *hTERT*-promoted Int-activation of the binary system in HEK293T cells (**A**) and in LLC-Kat cells (**B**), each compared with that of a *CMV*-promoted Int system. The data represent the mean value of three experiments; the error bars indicate standard deviation. RSL represents the *attR*-Stop-*attL* cassette (Fig. 1A).

Our first goal was to validate the efficiency of the binary cell expression system activated by Int in cancer cells. To this end we employed the immortalized HEK293T cells that are transformed both by adenovirus and by the SV40 large tumor antigen. This line expresses a high level of *hTERT* sufficient to maintain their telomeres indefinitely, unlike normal human cells that do not express *hTERT*^21^,^22^. Therefore, placing *int* under the control of *hTERT* promoter was expected to express in the HEK293T cells. HEK293T cells were co-transfected with the silent *luc* substrate carrying the *CMV*-*attR*-Stop-*attL*-*luc* cassette (pAE1855, Fig. 1) and a plasmid expressing *int* either under the control of the cancer specific *hTERT* promoter (pNA1263) or under the constitutive *CMV* promoter (pNA979) that served as a positive control. The quantitative analysis of the Luc activity assay in HEK293T cells (Fig. 2A) indicated that *int* driven by the *hTERT* promoter catalyzed *attR* x *attL* excision at ~20% the extent of that observed with the *CMV* control. In the absence of *int* Luc activity was negligible. Thus, *int* expressed from the cancer specific *hTERT* promoter catalyzed rather efficiently site-specific recombination in the immortalized HEK293T cells.

### A lung-specific DNA delivery in mice

**Figure 3:**
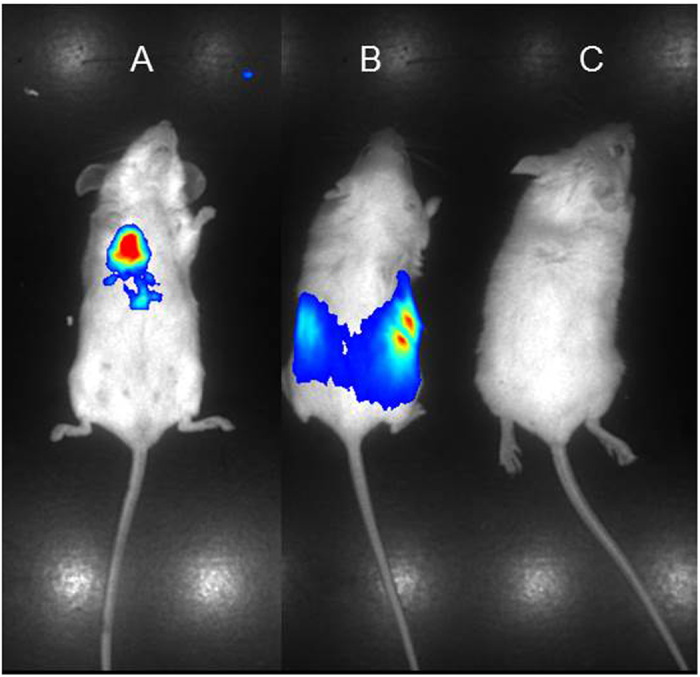
(**A**) Delivery of a *CMV*-*luc*-carrying plasmid (pYM1600) complexed with the *in vivo*-jet PEI into the lungs of an IV tail-injected BALB/c mouse. (**B**) Luc activity in a BALB/c *luc*-transgenic mouse. (**C**) Non-injected BALB/c mouse treated with luciferin.

Efficient *in vivo* transformation and gene delivery in mammals is a considerable technical challenge. Among different approaches and DNA delivery agents, including lentiviral vectors, we have observed that the linear *in vivo*-jet PEI (Polyplus, France) is the most effective. This linear polyethylenimine reagent is used to deliver DNA, siRNA and oligonucleotides. It forms stable complexes with the nucleic acid cargo, protecting it from degradation thereby facilitating *in vivo* delivery. These complexes pass the capillary barrier in the lung, explaining the propensity of *in vivo*-jet PEI to deliver its cargo specifically into lungs^23^. As reporter, we found that the Luciferase gene (*luc*), whose product emits characteristic yellow-green bioluminescence in the presence of its luciferin substrate is most suitable. BALB/c mice were intravenous (IV) tail-injected with a sample of the jet PEI delivery agent complexed with a reporter plasmid (pYM1600) that carries the *luc* gene under the control of *CMV* promoter. 24 hours later the mice were injected with the Luciferase substrate luciferin and underwent bioluminescence imaging. The treated mouse (Fig. 3A) was compared with a luciferin-injected *luc*-transgenic mouse as a positive control (Fig. 3B) and a BALB/c mouse injected with the luciferin substrate alone as a negative control (Fig. 3C). The treated mouse injected with the gene delivery system (Fig. 3A) showed specific bioluminescent activity in the lung area while the transgenic mouse (Fig. 3B) showed abdominal activity. We concluded that the *in vivo*-jet PEI carried the *luc* plasmid to the lungs and therefore we set out to develop lung cancer mice as an *in vivo* model to explore our Int activated cancer specific binary system *in vivo.*

### Int activates the *luc* reporter binary system in the lungs of BALB/c mice

**Figure 4:**
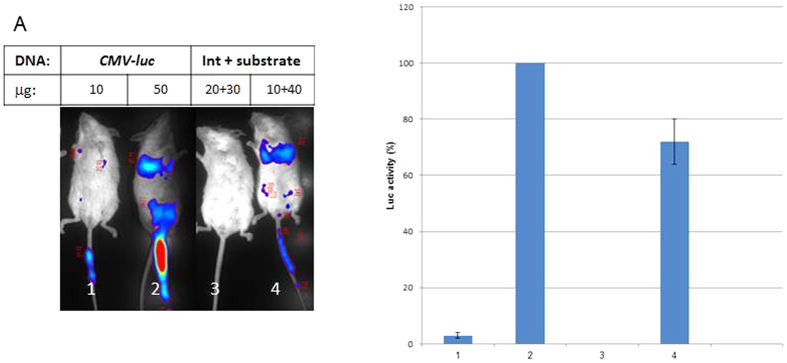
Int activity in the lungs of BALB/c mice. (**A**) Bioluminescence analysis. BALB/c mice were IV-injected with the *in vivo*-jet PEI complexed with indicated amounts of a *CMV*-*luc* plasmid as control (mice Nos 1 and 2) or with the complexed binary system (Nos 3 and 4). (**B**) Quantitative analysis of the bioluminescence data in the lung areas. The bars show the mean value of three experiments; the error bars indicate standard deviation.

We next tested if our binary system is also active *in vivo* in mice as it does in cell culture. Two mice were IV-tail injected with jet PEI complexed with different doses of the constitutive *CMV*-*int* expressing plasmid, and the silent *attR*-Stop-*attL-luc* substrate plasmid (20 μg + 30 μg and 10 μg + 40 μg respectively). As a positive control two mice were each similarly treated with two doses of a *CMV*-*luc* expressing plasmid (pYM1600, 50 μg and 10 μg). 24 hours later bioluminescence imaging of these mice (Fig. 4A) has shown that in mouse No. 4 injected with the higher dose of the silent substrate along with the lower dose of the Int expressing plasmid Int has catalyzed the recombination reaction that lead to the activation of the silent *luc* reporter in the lungs’ area (Fig. 1). Quantification of these assays (Fig. 4B) shows that the efficiency of the binary reaction is comparable to that acquired by control mouse No. 2 injected with higher dose of a *CMV*-*luc* expressing plasmid. These results show that under a constitutive *CMV*-*int* expression the binary system is also active *in vivo* in the mouse lungs. The sensitivity of Luc detection strongly depends on the concentration ratios of the injected plasmid, explained by the virtual absence of a positive signal in mice Nos 1 and 3.

### An experimental lung cancer mice model

Having demonstrated that the jet PEI DNA delivery reagent has delivered the *luc* plasmid exclusively to the lungs, our next step was to develop a mouse lung cancer model. To this end we used the Lewis lung carcinoma line 1 (LLC1)^24^ labelled with a far-red mutant of the red fluorescent gene of the sea anemone *Entacmaea quadricolor* known as Katushka cells (LLC-Kat)^25^,^26^. LLC1 cells remain highly tumorigenic in C57BL/6 mice and produce primary tumors and lung metastases. LLC1 cells are widely used as a model for the development of lung-metastatic tumors in mice and are useful in studying the mechanisms of cancer chemotherapeutic agents^27^. The presence of the Katushka label in LLC1 cells facilitates the detection of the lung metastases. A preliminary experiment with these cells shows that the binary system in these cells works as efficiently as in HEK293T cells (Fig. 2B).

**Figure 5:**
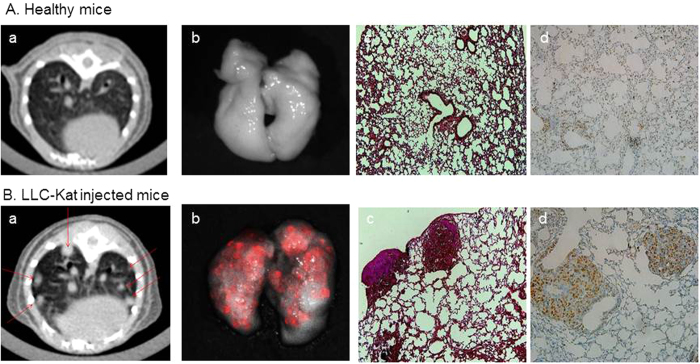
*In vivo* imaging and immunohistochemistry analysis of lungs from healthy (**A**) and LLC-Kat injected C57BL/6 mice (**B**). Panels (a), *in vivo* imaging using a micro-CT device. Arrows mark metastases location; panels (b), *ex vivo* fluorescence imaging of the lungs using a Maestro system; panels (c), H&E staining; panels (d), immunostaining for PCNA.

Lung tumor metastases development was examined nine days following the injection of the LLC-Kat cells using *in vivo* micro-CT scanning. No tumors were detected in the lungs of the non-injected control [Fig. 5A(a)] whereas in the injected mice metastases were detected and localized in the lungs [arrows in Fig. 5B(a)]. The presence of metastases in LLC-Kat lungs were verified by *ex-vivo* fluorescence detection using the Maestro imaging system [Fig. 5B(b)] as compared to non-injected control [Fig. 5A(b). Further validation that the injected mice have acquired lung cancer were performed by H&E staining [Fig. 5B(c)] and by immunostaining using proliferating cancer nuclear antigen (PCNA) [Fig. 5B(d)] compared to control healthy lungs [Fig. 5A(c,d)]. In both cases the cancer metastases were evident. These data demonstrate the generation of a mouse lung cancer model using injection of LLC-Kat cells in C57BL/6 mice. Moreover the mice died after approximately 14 days post injections.

### Cancer specificity of the Int-based binary cell expression system in mice

**Figure 6:**
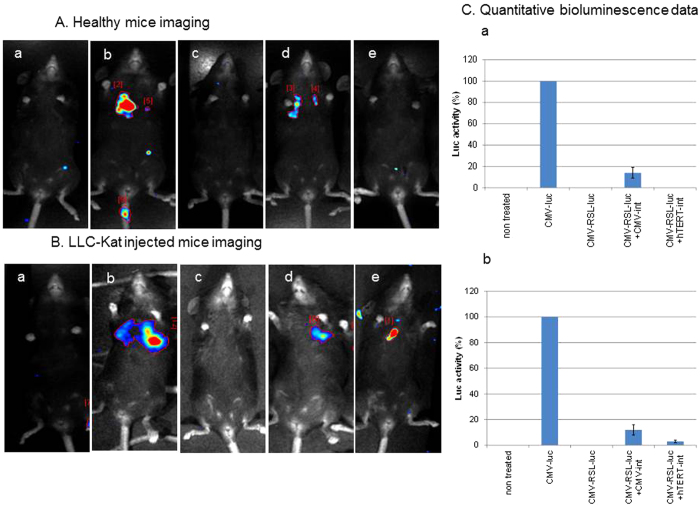
Cancer specificity of the binary *hTERT*-*int* based expression system using a cancerous mouse model. (**A**) Healthy C57BL/6 mice, (**B**) LLC-Kat injected counterparts. Panels (a), untreated mice; panels (b–e), IV-tail injected mice with the following plasmids complexed with the *in vivo*-jetPEI (b) *CMV*-*luc*, (pYM1600 - 50 μg) (c) *CMV*-RSL-*luc,* (pAE1855 - 40 μg) (d) *CMV*-RSL-*luc*, (pAE1855 - 40 μg) + *CMV*-*int*, (pNA979 - 10 μg) (e) *CMV*-RSL-*luc*, (pAE1855 - 40 μg) + *hTERT*-*int*, (pNA1263 -10 μg). The mice were injected 24 hr later with luciferin and their bioluminescence visualized as detailed in the Methods section. Each figure shows a typical mouse from a cohort of at least four mice. (**C**) Quantitative bioluminescence data. The bars show the mean value of four experiments; the error bars indicate standard deviation. RSL represents the *attR*-Stop-*attL* cassette.

**Figure 7:**
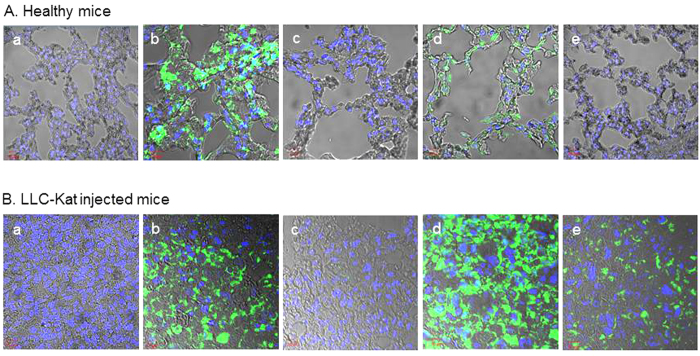
Immunohistochemical analyses of Luc expression in the lungs of the healthy mice (**A**) and of the LLC-Kat injected mice (**B**). The mice were treated in the same order as in Fig. 6. Each figure shows a typical mouse from a cohort of at least four mice.

Finally, to test the cancer specificity of the binary Int-activated cell expression system in mice we performed a comparative analysis of this system between healthy C57BL/6 mice (Fig. 6A) and LLC-Kat-infected mice (Fig. 6B). In each of these two types of C57BL/6 mice, there were four groups (4 mice/group) that were IV tail-injected with the *in vivo*-jet PEI carrier complexed with different combinations of plasmids [panels (b–e)] while groups (a) remained untreated. In groups (b) the jet PEI carrier was complexed with the *CMV*-*luc* reported as positive control; in groups (c) the carrier was complexed with the silent recombination substrate (*CMV*-RSL-*luc*) alone to validate the absence of luc leakage; in groups (d,e) the carrier was complexed with the silenced substrate and the *CMV*-promoted Int (d) or with the *hTERT*-promoted Int (e). 24 hours later all mice were injected with luciferin and underwent bioluminescence imaging. As expected, in both healthy and cancerous mice the negative controls (a) and the ones treated with the silent substrate (c) showed no bioluminescence while the positive controls (b) did. So did the two groups (d) treated with the silent substrate along with the constitutive *CMV*-Int. However, in groups (e) only the cancerous mice treated with the silent substrate along with the *hTERT*-Int showed bioluminescence [Fig. 6B(e)] while in the healthy mice [Fig. 6A(e)] no bioluminescence was evident. Quantitative data of the bioluminescence mice are shown in Fig. 6C. Though in the cancerous mice the bioluminescence that was specifically catalyzed by the activity of *hTERT*-Int was lower compared to the constitutive *CMV*-Int, it was consistent with the expected specificity of our binary cancer cell expression system. To reinforce this cancer specific effect, the treated healthy and cancer mice underwent an immunohistochemical analysis of Luc expression (Fig. 7). The expected negative controls [panels (a,c)] did not show any immune response, the positive treatments [panels (b,d)] showed an immune response in both types of mice, while among the mice treated with the *hTERT*-Int binary system [panels (e)] only the cancerous ones showed an immune response. These results unambiguously confirmed the cancer specificity of our binary system.

### Int does not show a toxic effect

**Figure 8:**
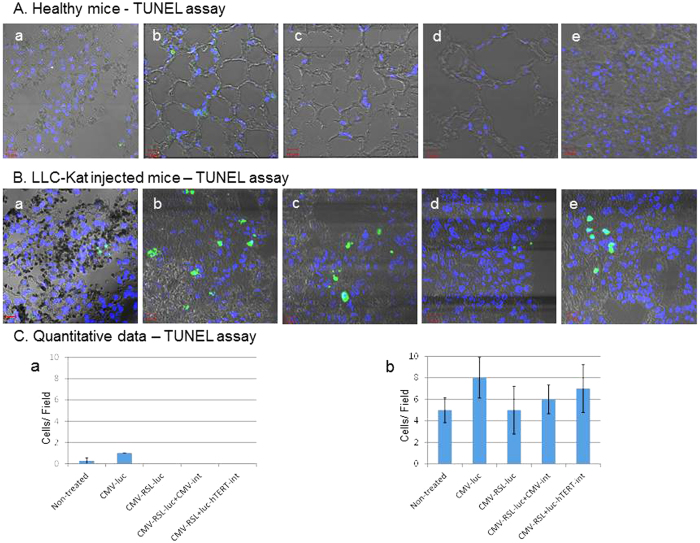
Immunohistochemical analysis of the TUNEL assays in the lungs of the healthy mice (**A**) and of the LLC-Kat injected mice (**B**) shown in the same order as in Fig. 6. Each figure shows a typical mouse from a cohort of at least four mice. (**C**) Quantitative data of the TUNEL assays in the lungs of the healthy mice (a) and of the LLC-Kat injected mice (b). The bars show the mean value of four experiments; the error bars indicate standard deviation.

Cre is one of the most widely SSRs used in mammalian gene manipulations, however, Cre-dependent cellular toxicity has been reported in eukaryotic cells and organisms some even under transient conditions. These include reduced cell growth, chromosome aberrations, brain development, cardiac fibrosis, heart failure, thymic atrophy and severe hematological toxicity^28^,^29^,^30^,^31^,^32^. To examine if, under the conditions of the above treatments, Int has any toxic effect we performed a TUNEL assay^33^ on the lungs of the same treated and untreated mice, thereby evaluating the apoptotic effect of the binary system. As evident from Fig. 8A all healthy lungs showed only negligible or no levels of apoptosis while the cancer lungs (Fig. 8B) were apoptotic as expected in those types of cells^34^. However, neither the lungs of the healthy mice treated with Int [Fig. 8A(d,e)] showed any apoptosis nor did their parallel cancer lungs [Fig. 8B(d,e)] show any increase in apoptosis relative to the ones in the absence of Int [panels (a,b)]. Quantitative data of the TUNEL assay confirm these observation (Fig. 8C), concluding that under our experimental conditions the amounts of supplied Int had no toxic effect.

## Discussion

In the present study a new cancer specific binary cell expression system was developed that is based on the cancer-specific *hTERT* promoter showing that when the expression of coliphage HK022 Int is promoted by *hTERT* it activates, by a site-specific recombination reaction, the expression of the *luc* reporter gene exclusively in lung cancer cells without affecting normal tissues. In previous two reports that employed the Cre/*lox* system in such binary systems one used an adenovirus vector as the gene delivery carrier^10^ and the other combined the two binary elements (recombinase and RS) by crossing two transgenic mice, each carrying one of them^9^. The advantages of our system are that it is not involved with a risky mammalian viral DNA carrier, that it can be delivered transiently and that under the experimental conditions Int does not show any toxicity. However, to test if Int may have some toxic effect a more extensive study that uses dose calibration and more than one toxicity assay are still missing.

When using a toxin gene to be specifically expressed in cancer cells it is essential that in the binary system the recombinase, being an enzyme, be promoted by a weak and specific promoter and that the toxic gene of the substrate be completely blocked by a tight transcription terminator. Because more substrate than enzyme needs to be delivered (Fig. 4A) the direct use of an *hTERT* promoted substrate, though being cancer specific, may be somewhat leaky on its voyage from its delivery spot to the cancer tissue thereby loosing specificity by damaging healthy tissues. Admittedly, a successful direct adenoviral carrier delivery of an *hTERT* promoted DTA toxin that cured ovary cancer in mice was reported^35^, nevertheless extra caution is always an advantage when it goes with cancer therapy.

With regards to possible off-target chromosomal integration events catalyzed by Int, we have previously shown that for Int to promote integration into secondary *attB* sites, such sites need to to carry a perfect wild type 7 bp overlap with two flanking palindromic sequences^18^. Moreover, the *attL* and *attR* RS sites in the binary system are incomplete *attP*s. These and the fact that Int is weakly promoted by *hTERT* reduce the likelihood of off target Int-catalyzed integration events into human secondary *attB* sites.

In the Int-catalyzed binary system we employ the excisive *attL* and *attR* as recombination sites because they proved more efficient in mammalian recombination activity than does the *attB* + *attP* pair. Another reason is because their recombination leads to the short 21 bp *attB* site located between the promoter and the Luc gene (Fig. 1) that does not interfere with the expression of Int.

Preliminary experiments in cell culture with the binary system that carry subunit A of the diphtheria toxin (DTA) have already shown that with the DTA toxicity is clearly specific in the LLC-Kat as compared to normal human foreskin BJ cells and experiments with live mice using DTA and other toxins to eradicate the lung cancer are in progress. Success may render the system useful with other cancer cases using relevant cell specific promoters and cell specific DNA carries.

## Methods

### Cells, growth conditions, mice, plasmids and oligomers

The bacterial host used was *E. coli* K12 strain TAP114 (*lacZ*)M15^36^. It was grown and plated on Luria-Bertani (LB) rich medium with the appropriate antibiotics. Plasmid transformations were performed by electroporation^37^. Plasmids and oligomers are listed in Tables 1 and 2, respectively.

Human embryonic kidney cells HEK293T (ATCC CRL-3216) were cultured in Dulbecco’s modified Eagle’s medium. Mouse Lewis lung carcinoma LLC1 (ATCC CRL-1642) cells labelled by Katushka fluorescent gene reporter^26^ were cultured in RPMI medium.

For transient transfection of HEK293T and LLC-Kat, the cells (~6×10^5^) were plated in a 6 well plate and 24 hours later treated with 2 μg circular plasmid DNA using Puro-Fection transfection reagent (System Biosciences, USA) or TransIT-XT2 reagent (Mirus, Madison, WI, USA), respectively.

All mice procedures were performed in compliance with Tel Aviv University guidelines and protocols approved by the Institutional Animal Care and Use Committee. BALB/c and C57BL/6 strains were used for the mice experiments. In experiments involving injected LLC-Kat cells we used only the C57BL/6 strain.

All experiments described below were at least triplicated.

### Plasmid construction

The pNA1263 plasmid (Table 1) carrying the int gene of HK022 under the control of *hTERT* promoter (*hTERT)* was constructed as follows. A BglII-HindIII *hTERT* PCR fragment, was generated using primers oEY638 + oEY639 (Table 2) and an *hTERT*-plasmid as template provided by Dr. S. C. Teng at the Department of Microbiology, National Taiwan University., and this PCR product was ligated into the matching sites of plasmid pNA979^38^. The pYM1600 plasmid, carrying the *luc* gene under the control of *CMV* promoter was constructed by the RF cloning procedure^39^. The *CMV* fragment that was obtained by PCR using plasmid pCDNA3 as a template and primers oEY843 + 850 was introduced into the luciferase reporter vector pGL2 vector (Promega, Madison, WI, USA). The pAE1855 plasmid, used as a substrate for the *CMV*-Luc assays, was constructed by ligation of a AgeI-NotI *luc* PCR fragment, obtained using pYM1600 as template and primers oEY807 + 808, into the same sites of the intermediate plasmid construct pAE1855inter carrying *CMV*- *attR*-Stop-*attL* cassette on pCDNA3. All plasmid constructs were verified by DNA sequencing.

### Luc activity assay in cell culture

HEK293T or LLC-Kat cells transfected by the appropriate *luc* plasmids were collected 48 hours later by trypsin treatment and centrifugation, resuspension in 100 μl of x1 Passive Lysis Buffer (Promega, Madison, WI, USA) followed by sonication and centrifugation. Quantitative Luc analysis of the supernatant was performed using Dual-luciferase reporter assay kit (Promega, Madison, WI, USA) and Synergy2 plate reader (Biotek, USA) according to the manufacturer’s instructions.

### DNA delivery and Luc activity imaging in mice

BALB/c or C57BL/6 mice were IV tail-injected with 250 μl of the *in vivo*-jet PEI delivery reagent (Polyplus transfection, France) complexed with an appropriate *luc* reporter plasmid. 24 hours later the mice were injected with 250 μl of 3 mg/ml luciferin solution (Gold Biotechnology, USA) and underwent bioluminescence imaging by Biospace Photon Imager (Biospace Lab, France).

### Lung cancer development and fluorescence imaging in mice

0.8 × 10^6^ LLC-Kat cells were IV tail-injected into 8 week old (17–21 gr) female C57BL/6 mice. Lung tumor metastases development was examined nine and twelve days following the injection using *in vivo* micro-CT scanner (TomoScope® *In-vivo* CT, Germany). The Katushka fluorescent signal in the lung metastases was analyzed on day 12 by an *in vivo* fluorescence imaging system (CRi Maestro^TM^, USA).

### Preparation of Sections, Immunohistochemistry, Confocal Microscopy, apoptosis TUNEL assay and Image analysis

At the end of the experiments, biopsy specimens from lungs were collected, fixed in 4% paraformaldehyde (PFA), embedded in paraplast and 5 μm sections were prepared. Healthy and cancer lungs carrying metastases were treated in parallel with H&E stained and immune-stained anti-Luciferase antibody (Abcam, Cambridge, UK) at 1:100 dilution, with a PCNA kit (Life Technologies, Carlsbad, CA, USA) and with a TUNEL kit (MBL international, Woburn, MA, USA) according to manufacturer’s instructions. Donkey anti-Goat IgG (H + L) secondary antibodies conjugated with Alexa Fluor 488 dye (Life Technologies, Carlsbad, CA, USA) were used for Luciferase detection. Nuclei were detected with DAPI (Biolegend, San Diego, CA, USA). At least 20 images of five tissue sections from 3 different mice per group were obtained for each analysis. Microscopic observation and image acquisition were performed using a Zeiss 510 Meta – NLO confocal laser-scanning microscope (Zeiss, Oberkochen, Germany). TUNEL assay analysis was performed using Image J software.

### DNA manipulations

Plasmid DNA from *E. coli* was prepared using a DNA Spin Plasmid DNA purification Kit (Intron Biotechnology, Korea) or a NucleoBond^TM^ Xtra Maxi Plus EF kit (Macherey-Nagel, Germany). General genetic engineering experiments were performed as described by Sambrook and Russell^37^.

## Additional Information

**How to cite this article**: Elias, A. *et al.* Cancer-specific binary expression system activated in mice by bacteriophage HK022 Integrase. *Sci. Rep.* **6**, 24971; doi: 10.1038/srep24971 (2016).

## Figures and Tables

**Table 1:** List of plasmids.

Plasmid	Relevant genotype*	Source
pNA979	*CMV*-*int*	38
pNA1263	*hTERT*-*int*	this work
pYM1600	*CMV*-*luc*	this work
pAE1855inter	*CMV*-*attR*-Stop-*attL*	this work
pAE1855	*CMV*-*attR*-Stop-*attL*-*luc*	this work
pcDNA3	Neo^R^ Ap^R^	Invitrogen
pGL2	*luc* Ap^R^	Promega

**Table 2:** List of oligomers.

Primer	Sequence	Locus*
oEY638	GGAGATCTGGATTCGCGGGCACAGAC	*hTERT*
oEY639	CCAAGCTTCCAGGGCTTCCCACGTG	*hTERT*
oEY807	GGACCGGTATGGAAGACGCCAAAAACATAAAGAAAGGC	*luc*
oEY808	GGGCGGCCGCTTACAATTTGGACTTTCCGCCCTTCTTGG	*luc*
oEY843	GCTAACATAACCCGGGAGGTACCGAGCTCTCTGTGGATAACCGTATTACCGCCATGC	*CMV*
oEY850	CAACAGTACCGGAATGCCAAGCTTACTTAGGCGCTAGCGGATCTGACGG	*CMV*
